# *In vitro* assessment of inter and intra batch variability of a breath-enhanced jet nebulizer

**DOI:** 10.1186/2045-7022-3-S1-P11

**Published:** 2013-05-03

**Authors:** Mariam Rehman, Antony Hurren, Adam Metcalf, Ross Hatley

**Affiliations:** 1 Respironics Respiratory Drug Delivery (UK) Ltd, Philips Home Healthcare Solutions, UK

## Background

Guidelines recommend that patients presenting with acute severe asthma with life-threatening features receive the necessary high doses of β2-agonists via the nebulized route.The SideStream Plus (SS+; Philips Respironics) is a breath-enhanced nebulizer designed to maximize respirable output, while minimizing treatment time [1]. Prior to the introduction of a new version of nebulizer, various *in vitro* assessments, including inter and intra batch variability, are performed using a limited number of devices. We present results of an analysis of inter and intra batch variability of SS+nebulizers.

## Method

SS+ nebulizers (3 batches; n=30 per batch) were assessed in terms of 5 experimental parameters; nebulization time, residual volume, particle size distribution (mass median diameter (MMD), fine particle fraction (FPF)), and emitted dose. Each batch was provided with a driving flow from a different Porta-neb compressor (Philips Respironics). Measurement of particle size distribution was achieved using a laser diffractor (Malvern Spraytec) with an extraction flow of 15 l/min. Each nebulizer was weighed and filled with 2.5 ml salbutamol sulphate (albuterol sulfate) solution (1 mg/ml). To assess nebulization duration the nebulizers were run continuously until ‘end of treatment’, defined as when the obscuration level of the sample in the laser diffractor fell below 5% for 5 s. At this point the SS+ were re-weighed to calculate residual volume. The dose delivered to a filter placed between the laser diffractor and extraction air flow was quantified using high performance liquid chromatography. The coefficient of variation (CV) for each experimental parameter was calculated for each batch.

## Results

Results are shown in Table1.

**Table 1:** 

	Batch A CV (%)	Batch A mean	Batch B CV (%)	Batch B mean	Batch C CV (%)	Batch C mean
Nebulization time (s)	13.1	274	9.3	258	9.6	270
Residual volume (mg solution)	6.6	1035	8.1	1100	9.9	1086
MMD (µm)	2.9	3.8	3.1	3.9	3.9	3.8
FPF (%<5 µm)	2.1	63.2	2.3	62.6	3.0	63.4
Emitted dose (µg salbutamol)	6.9	1127	9.2	1125	8.8	1101

## Conclusion

The intra batch coefficient of variation indicates that variability in each of the 5 experimental parameters did not differ substantially across the 3 batches tested. The close similarity between mean values for each of the experimental parameters indicates that inter batch variability was low. Taken together, these results suggest that different batches of SS+ nebulizers should perform similarly in terms of nebulization time, particle size distribution, and emitted dose.
